# Inside the head of *Crotalus durissus* LINNAEUS, 1758 (Serpentes, Viperidae, Crotalinae): Macroscopic description of the brain with ontogenetic insights

**DOI:** 10.1002/ar.25672

**Published:** 2025-04-16

**Authors:** Giordanna Issa Lucas, Angele Martins

**Affiliations:** ^1^ Laboratório de Anatomia Comparada dos Vertebrados, Departamento de Ciências Fisiológicas, Instituto de Ciências Biológicas Universidade de Brasília Asa Norte Brasília, DF Brazil; ^2^ Setor de Herpetologia, Departamento de Vertebrados Museu Nacional/UFRJ Rio de Janeiro RJ Brazil

**Keywords:** intraspecific variation, morphology, neuroanatomy, ontogeny, rattlesnake

## Abstract

Neuroanatomy studies in vertebrates have garnered significant attention in recent years, particularly driven by advancements in computerized tomography imaging techniques. Nonetheless, these advancements remain largely constrained to specific vertebrate groups, notably mammals, birds, and fish, leaving studies in reptiles at an incipient stage. In this work, we aim to describe in detail the macroscopic morphology of the brain of *Crotalus durissus* based on a sample of four young and four adult individuals—three male and five female specimens, providing the first detailed description of the brain with a relatively modest sample available for reptiles. Our results show that the major macroscopic features identified in *C. durissus* suggest a brain structure typical of a multi‐habitat and cathemeral/nocturnal alethinophidian species, thereby contributing significant data to the understanding of brain morphological evolution in snakes. Brain measurements showed distinct scaling patterns related to snout‐vent length and head length, with variables such as brain length and cerebral hemisphere length decreasing with SVL, while others like olfactory bulb length and medulla oblongata width increased. Additional differences were observed comparing juveniles and adults, with adults generally exhibiting larger mean values for cerebellum and medulla oblongata measurements. However, the small sample size highlights the need for future studies with larger datasets to validate these findings and explore the developmental trajectories in greater detail.

## 1 INTRODUCTION

The brain represents the main organ in the central nervous system of vertebrates, integrating physiological and environmental sensory information for homeostasis (Scanferla, 2022). In the last decades, studies on evolutionary biology and behavior have garnered increasing interest, triggered by a series of investigations assessing potential brain evolution similarities across a diverse set of taxa (e.g., Abbott et al., 1999; Amiel et al., 2011; de Winter & Oxnard, 2001; Safi & Dechmann, 2005). Such studies have been particularly important in pointing out that interspecific differences in brain region size are associated with variations in ecological, evolutionary, and systematic aspects among vertebrates.

Much of the recent progress in neuroanatomical studies has been facilitated by the advent of contrast‐enhanced computed tomography (CT) imaging, which provides a satisfactory level of detail (e.g. Allemand et al., 2023; Watanabe et al., 2019) as an alternative to the use of destructive techniques such as manual dissection and/or histology. Computed tomography imaging has been especially interesting for fossil studies (e.g., Triviño et al., 2018; Macrì et al. 2023), also yielding a significant progress in neuroanatomical studies, since it allows new abilities to examine endocranial cavities through digital endocasts, including the reconstruction of brain endocasts of fossil forms (Scanferla, 2022). However, specific brain regions/structures—especially those extremely reduced such as the epiphysis, cerebellum, and pituitary gland—usually fail to be satisfactorily represented in brain endocasts (Scanferla, 2022; Watanabe et al., 2019). Thus, for descriptive purposes, dice‐CT scanning of the head and/or manual dissection of the brain are still the most recommended methods for describing the brain in detail (Scanferla, 2022).

General patterns associated with brain evolution have—among vertebrates—predominantly been studied using models such as fishes, birds, and mammals, with a special focus on major evolutionary issues (Kotrschal & Kotrschal, 2020; Powell & Leal, 2012; Loza et al., 2023). Compared to other clades, the patterns of brain evolution in amphibians and reptiles have largely been overlooked, with most of the available studies for reptiles concentrating on the relationship between brain and body size, or general evolutionary aspects for broader purposes, with very few key lineages explored (e.g., Allemand et al., 2023; Macri et al., 2019). Additionally, considering the few studies currently available, only a small number of contributions focus on the detailed description of the neuroanatomy of snake lineages, and, consequently, the brain anatomy of snakes is still incipient.

The suborder Serpentes is composed of about 4100 known snake species (Uetz et al., 2024), representing a highly diverse group of reptiles characterized by their remarkable adaptations and variations related to their morphology, ecology, and geographic distribution (Greene, 1997). Snakes are traditionally divided into two major lineages—“Scolecophidia” and Alethinophidia (Vidal et al. 2010; Zaher et al., 2019) with “scolecophidians” being characterized by their small size associated with body miniaturization, fossorial habits, and several morphological adaptations to burrowing (Greene, 1997; Martins et al., 2020, 2021); and alethinophidians comprising the so‐called “true snakes” representing a clade that includes both primitive (such as boas and pythons) and the “advanced snakes” (=Caenophidia) that display a range of adaptations and habitats (Greene, 1997; Cundall & Irish, 2008).

Despite the latest efforts in assessing the brain morphology of snakes, neuroanatomical data—especially those of gross morphology—remain extremely scarce despite the vast diversity of currently recognized species, and consequently, the anatomical diversity of the snake brain remains unclear (Scanferla & Smith, 2020; Scanferla, 2022). Since the seminal work of Senn (1966), very few studies have been conducted posteriorly with systematic and/or functional purposes (e.g., Masai et al., 1980; Scanferla, 2022). Sparse recent contributions have reinforced the utility of neuroanatomical data for several major evolutionary purposes, such as the ecological origin of snakes (Macrì et al. 2023), macroevolutionary patterns related to habitat use (e.g., Scanferla, 2022; Segall et al., 2021), functional aspects (e.g. Catania, 2011; Scanferla, 2022), and even for providing systematic insights among the lineages of the burrowing scolecophidians and alethinophidians (Scanferla, 2022), therefore reinforcing the importance of neuroanatomical data for this group.

Vipers (Caenophidia, Viperidae) represent a fascinating lineage of approximately 450 species of venomous snakes that occur throughout the globe, except for Antarctica, Australia, and other islands such as Madagascar, New Zealand, and Ireland (Uetz et al., 2024). The species belonging to Viperidae exhibit specialized sensory organs (thermoceptor pit organs) and are traditionally subdivided into three subfamilies—Viperinae, Azemiopinae, and Crotalinae (e.g., Hsiang et al., 2015; Zheng & Wiens, 2016; Zaher et al., 2019). Despite the several morphological studies that have accumulated in the past years, no detailed study has previously been conducted on the detailed descriptive neuroanatomy of representatives of Viperidae.

The genus *Crotalus* (Viperidae, Crotalinae) is currently composed of 43 species commonly known as rattlesnakes or rattlers (Uetz et al., 2024), with distribution restricted to the Americas, from southern Canada to Northern Argentina (Campbell & Lamar, 2004). The south american rattlesnake *Crotalus durissus* (Crotalinae) is a venomous pit viper species that occurs throughout Central and South America and inhabits a variety of environments, ranging from dry savannas to tropical forests, as well as open grasslands and shrublands (Campbell & Lamar, 2004; Wuster et al., 2005). The species is predominantly terrestrial, being mostly active during nocturnal hours, though it can be crepuscular depending on specific environmental conditions (Tozetti & Martins 1993).

Herein, we provide the first detailed and comparative macroscopic anatomical description of the brain of *C. durissus* based on a sample of eight individuals, including juveniles and adults. Thus, we provide relevant data on ontogenetic variation of the brain for the species and discuss possible allometric patters associated with growth.

## 2 MATERIALS AND METHODS

We manually dissected eight individuals (four young and four adults) from the collection of the Laboratório de Anatomia Comparada de Vertebrados (LACV) and Laboratório de Fauna e Unidades de Conservação (LaFUC), UnB (see Material examined for data). Prior to the dissection, we measured the snout‐vent length (SVL) and total length of each specimen with a flexible ruler. head length (HL), head width (HW), and head height (HH) of the individuals were measured using a Mitutoyo dial caliper to the nearest 0.5 mm. We identified males based on the confirmation of the presence of hemipenes after a ventral incision on the subcaudals, while juveniles and adults were identified based on Marinho (2007).

Manual dissection for brain removal consisted of the removal of the head skin and adjacent muscles with the aid of a scalpel and tweezers. Subsequently, the dorsal and lateral bone elements of the skull were removed, thus exposing the brain dorsally. At a later stage, the brain was removed from the remaining braincase through the section of the spinal cord and preserved in 70% ethanol. Several nerves were damaged upon dissection and will not be mentioned in this study.

**FIGURE 1:**
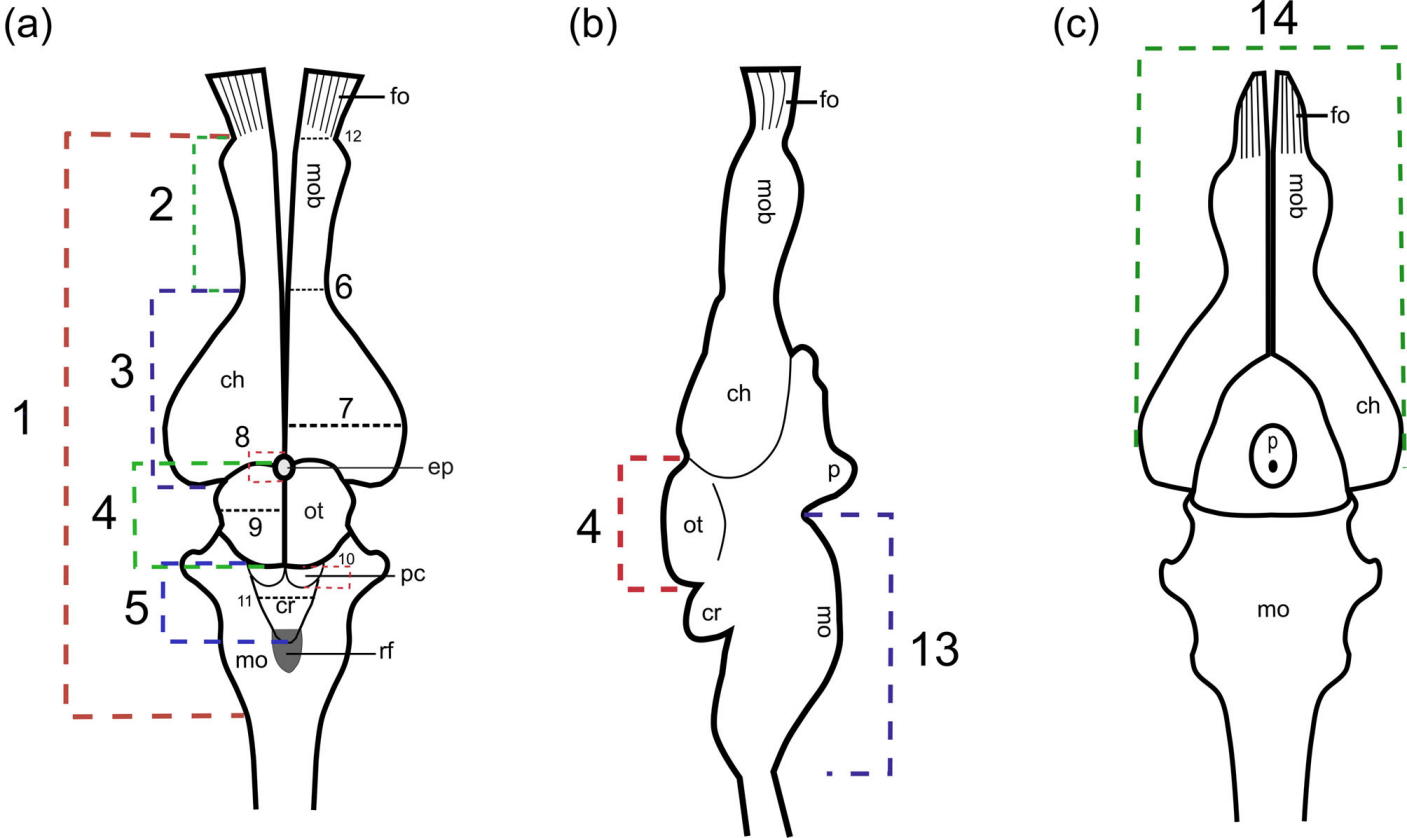
Schematic drawing in dorsal, lateral, and ventral views of the brain of *Crotalus durissus* with identification of linear measurements taken. Ch, cerebral hemisphere; cr, cerebellum; ep, epiphysis; fo, fila olfactoria; mob, olfactory bulb; mo, medulla oblongata; ot, optic tectum; p, pituitary; py, pyramid; rf, rhomboid fossa. Measurements: Brain length (1), olfactory bulb length (2), cerebral hemisphere length (3), optic tectum length (4), cerebellum length (5), olfactory bulb width (6), cerebral hemispheres width (7), epiphysis length (8), optic tectum width (9), posterior colliculi length (10), cerebellum width (11), fila olfactoria width (12), medulla oblongata length (13), brain width (14).

Linear measurements of 15 specific regions or brain structures were performed using the FIJI program version 2.3.0 and are demonstrated in Figure 1. Measurements are as follows: brain length (BL), brain width (BW), olfactory bulb length (OBL), olfactory bulb width (OBW), cerebral hemisphere length (CHL), cerebral hemispheres width (CHW), optic tectum length (OTL), optic tectum width (OTW), medulla oblongata length (MOL), medulla oblongata width at largest point (MOW), cerebellum length (CL), cerebellum width (CW), epiphysis length (EL), posterior colliculi length (PCL), and *fila olfactoria* width (FOW). For descriptive purposes, the size of brain structures/regions is demonstrated as a percentage of the total length or total width of the brain (see Figure 1); and the percentage of the brain is demonstrated in relation to those of the head prior to brain removal.

Graphs were made using R and R studio with a ggplot2 package (version 3.4.2). Scatter plots were generated to visualize the relationship between SVL and brain‐related variables, with fitted trend lines (linear regression) added to illustrate overall patterns. Boxplots were used to compare brain measurements between juvenile and adult groups, highlighting differences in distributions and variability. Nomenclatures follow Scanferla (2022).

### 2.1 Material examined

*C. durissus* (*n* = 8). BRAZIL. Distrito Federal: LACV 4327^1^. Unknown locality: LACV 3360^1^, LACV 3361^1^*, LACV 3362^1^*, LAFUC 02768^2^*, LACV 4323*^2^, LACV 4324^1^, LACV 4325^2^, LACV 4327^2^ (legends: *juvenile individuals;^1^ Female; ^2^Male).

## 3 RESULTS

### 3.1 General aspect

**FIGURE 2:**
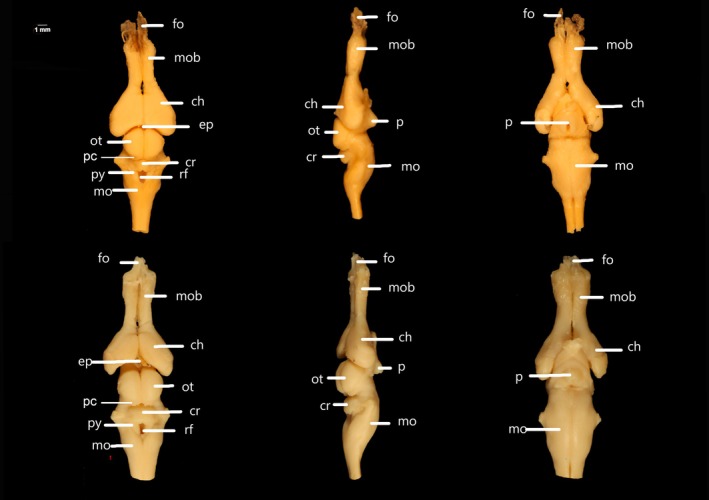
Dorsal, ventral, and lateral views of the brain of adult individuals of *Crotalus durissus* (LACV 4323 and LACV 4324). Ch, cerebral hemisphere; cr, cerebellum; ep, epiphysis; fo, fila olfactoria; mo, medulla oblongata; mob, olfactory bulb; ot, optic tectum; p, pituitary; py, pyramid; pc, posterior colliculi; rf, rhomboid fossa.

**FIGURE 3:**
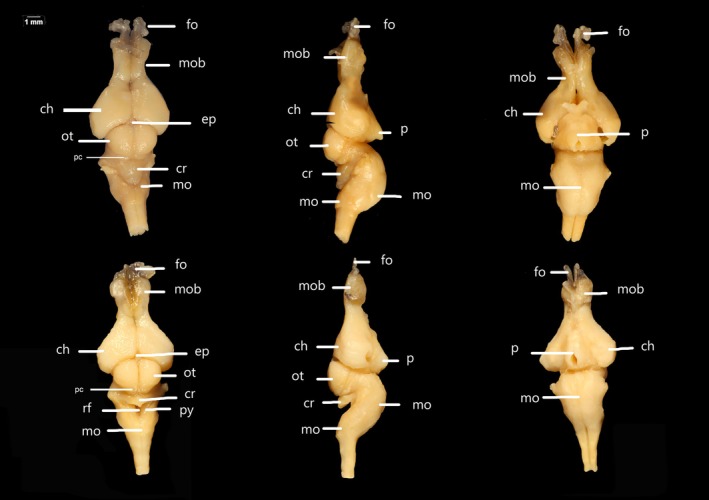
Dorsal, ventral, and lateral views of the brain of juvenile individuals of *Crotalus durissus* (LACV 3360 and LACV 3362). Ch, cerebral hemisphere; cr, cerebellum; ep, epiphysis; fo, fila olfactoria; mo, medulla oblongata; mob, olfactory bulb; ot, optic tectum; p, pituitary; py, pyramid; pc, posterior colliculi; rf, rhomboid fossa.

**FIGURE 4:**
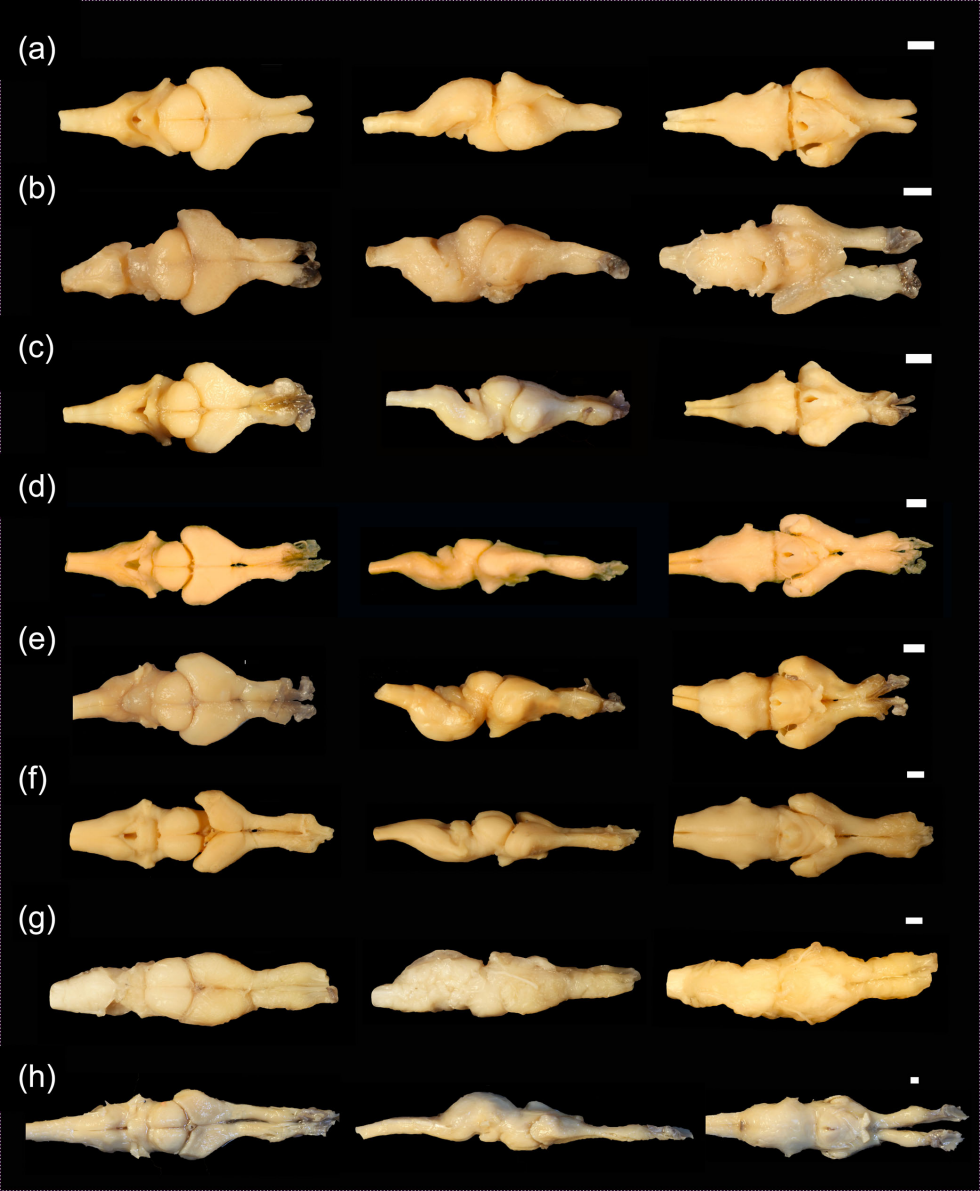
Dorsal, lateral, and ventral view (from left to right) of the brain of young and adult specimens of *Crotalus durissus* showing the variation through ontogeny. (a) LACV 3361 (330 mm SVL, female); (b) LAFUC 2768 (350 mm SVL, male); (c) LACV 3362 (380 mm SVL, female); (d) LACV 4323 (420 mm SVL, male); (e) LACV 3360 (SVL 740 mm, female); (f) LACV 4324 (905 mm SVL, female); (g) LACV 4327 (1110 mm SVL, female); (h) LACV 4325 (1180 mm SVL, female). Scale 1 mm.

The brain of *C. durissus* has an anteroposterior elongated pattern in adults and a relatively more robust pattern in young specimens (see Figures 2, 3, 4; Table 1). The dura mater and secondary meninge are visible upon removal of the dorsal skull elements, the latter being strongly adhered to the brain, particularly to the *fila olfactoria* (Figure 4g). In adults, the brain is three times as long as it is wide, whereas in young specimens it is only twice as long in relation to width. We did not find any qualitative or quantitative differences of the brain between young male and female specimens related to skull general shape and size. The total length of the brain varies between 50.3% in adults and 40% in young related to head length. Relative proportion (shown as %) of BL are as follows: the olfactory bulb of adults represents 24.6%, and 24.5% in young specimens; the hemispheres are 29% in adults and 32.5% in young; the optic tectum accounts for 18.2% in adults and 20.7% in young; the CL is 13% in adults and 8.55% in young specimens, and MOL, 37.6% and 36%, and width is 80% and 56.7% in adults and young respectively (see Table 1 for mean, standard deviation, minimum and maximum values).

**TABLE 1:** Qualitative data of the brain of young and adult individuals of *Crotalus durissus*.

	Young (330 mm–420 mm SVL)	Adult (740 mm–1180 mm SVL)
BL	40.4 ± 9.8 (29.1–52.6); *n* = 4	50.3 ± 11.6 (48.1–67); *n* = 4
BW	39.9 ± 6.7 (31.1–47,3); *n* = 4	25.1 ± 11.3 (14.3–40,7); *n* = 4
BL/BW	1.3 ± 0.2 (1–1.5); *n* = 4	1.8 (1–2.6); *n* = 4
B/HL	50.3 ± 11.6 (48.1–67); *n* = 4	40.4 ± 9.9 (29.1–52.6); *n* = 4
CHL	32.5 ± 1.9 (31.3–35.3); *n* = 4	29 ± 5.3 (22.3–35.3); *n* = 4
CL	8.55 ± 1.3 (8.3–10); *n* = 3	13 ± 2.3 (10.5–15.9); *n* = 3
CW	36 ± 7.5 (27.4–41.6); *n* = 3	37.6 ± 8.4 (31.14–50); *n* = 3
EL	3.4 ± 1.6 (2.3–4.6); *n* = 2	4.2 ± 1 (3.3–5.3); *n* = 3
MOL	41.7 ± 10.7 (35.1–57.6); *n* = 4	37.6 ± 7.6 (27.4–43.3); n = 4
MOW	56.7 ± 6.5 (51.8–66.2); *n* = 4	80 ± 16.9 (55.7–83.75); *n* = 4
OBL	24.5 ± 2.7 (22–28.2); *n* = 4	24.6 ± 2.8 (21.5–28.3); *n* = 4
OBW	18.4 ± 0.5 (17.7–18.8); *n* = 4	18.7 ± 1.4 (17.1–20.1); *n* = 4
OTL	20.7 ± 2.3 (18.5–23); *n* = 4	18.2 ± 1.8 (16–18.7); *n* = 4
OTW	32.3 ± 1.5 (30.9–33.6); *n* = 4	31 ± 3.7 (26.1 ± 33.9); *n* = 4
PCL	1.99 (*n* = 1)	2.5 ± 0.9 (1.6–3.3); *n* = 3

*Note*: Measurements are given in % of total head length or head width. All values are given as % of head length (brain length), head width (brain width), brain length (all other length measurements) or brain width (all other width measurements).

Abbreviations: BL, brain length; BW, brain width; BL/BW, ratio brain length/brain width, B/HL, ratio brain length related to head length; OBL, olfactory bulb length, olfactory bulb width (OBW), cerebral hemisphere length (CHL), optic tectum length (OTL); OTW, optic tectum width; MOL, medulla oblongata length; MOW, medulla oblongata width at largest point; CL, cerebellum length; CW, cerebellum width; EL, epiphysis length; PCL, posterior colliculi length, visible in dorsal view.

### 3.2 Forebrain

The forebrain (i.e., macroscopically visible structures) is composed of the paired olfactory bulbs, the cerebral hemispheres, the epiphysis, and the pituitary gland (Figures 2, 3, 4). While the olfactory bulbs are similar in length and width in young and adults (see Table 1), the cerebral hemispheres and epiphysis slightly differ in length between young and adults. The fila olfactoria (axons that enter the olfactory bulb region from the nasal sac and the vomeronasal organ, sensu Scanferla, 2022) (Figures 2, 3, 4) represents the anterior most portion of the forebrain, being visible dorsally, laterally, and ventrally. The total number of fibers at each *fila olfactoria* varies from three to five. The olfactory bulbs (see in Figures 2, 3, 4) have a dorsoventral cylindrical shape and are robust (*n* = 7) or elongated (*n* = 1). Each olfactory bulb connects posteriorly to each cerebral hemisphere. The portion that connects the posterior part to the hemispheres at their rostral ends is similar in width to the olfactory bulb, being the thinnest region of the brain. The cerebral hemispheres (Figures 2, 3, 4), in dorsal view, have a well‐defined medial sulcus and are laterally convex and medially straight. Ventrally, the cerebral hemispheres are bulky, more rounded, and laterally expanded in young specimens. The epiphysis, located dorsally to the brain, fits between the hemispheres and the optic tectum (Figures 2, 3, 4), representing the smallest visible macrostructure of the brain. This structure is prominent and spherical (*n* = 2) or diamond‐shaped (*n* = 1). However, in five individuals, it was damaged/not identified. The pituitary gland (Figures 1 and 2) is located ventrally, being semi‐circular (*n* = 5) or semi‐triangular (*n* = 3). This structure exhibits a medial opening and does not exhibit any level of ontogenetic or sexual dimorphism differences. In ventral view, the adenohypophysis of the pituitary gland was partially removed during the dissection.

### 3.3 Midbrain

The midbrain represents half the size of the cerebral hemispheres, located in the middle portion of the brain. In the dorsal view, the optic tectum is semi‐circular (*n* = 7) or spherical (*n* = 1), conspicuous, with well‐defined sagittal grooves. In adults, it is slightly shorter (18.2%) than in young specimens (20.7). The posterior colliculi (*n* = 7) dorsally cover the anterior most portion of the cerebellum and are almost fully covered by the optic tectum in dorsal view (see Table 1). Ventrally, this region is fused with the pituitary gland, with no distinction between the left and right sides of the lobes. In lateral view, the optic tectum is flat, despite being the highest part of the brain.

### 3.4 Hindbrain

The hindbrain lies posterior to the midbrain and is exclusively represented by the cerebellum and the medulla oblongata (see both in Figures 2, 3, 4). Its anterior most portion is overlapped by the posterior colliculi of the optic tectum. The cerebellum is visible only in dorsal view, being trapezoidal in shape (*n* = 7), with an inconspicuous longitudinal fissure, with its length representing 8.55% and 13% of the BL in young and adult, respectively.

The medulla oblongata constitutes the most posterior element of the brain, being elongated and semi‐triangular with a concave anterior end in adults (dorsal view), while in juveniles it is not elongated. In ventral view, the medulla oblongata is a single voluminous and well‐pronounced region. This structure is slightly longer in young (41.7%) in comparison with adults (37.6%), while it is narrower in width in young (56.7%) and wider in adults (80%).

The rhomboid fossa is triangular with a very tapered posterior portion (Figures 2, 3, 4), and the pyramids are narrow and short (*n* = 2). However, most rhomboid fossae are not detectable (*n* = 6). There is no overlapping of structures in adults, but in juvenile individuals the cerebellum covers the rhomboid fossa.

### 3.5 Ontogenetic variations and Allometric patterns of brain measurements

**FIGURE 5:**
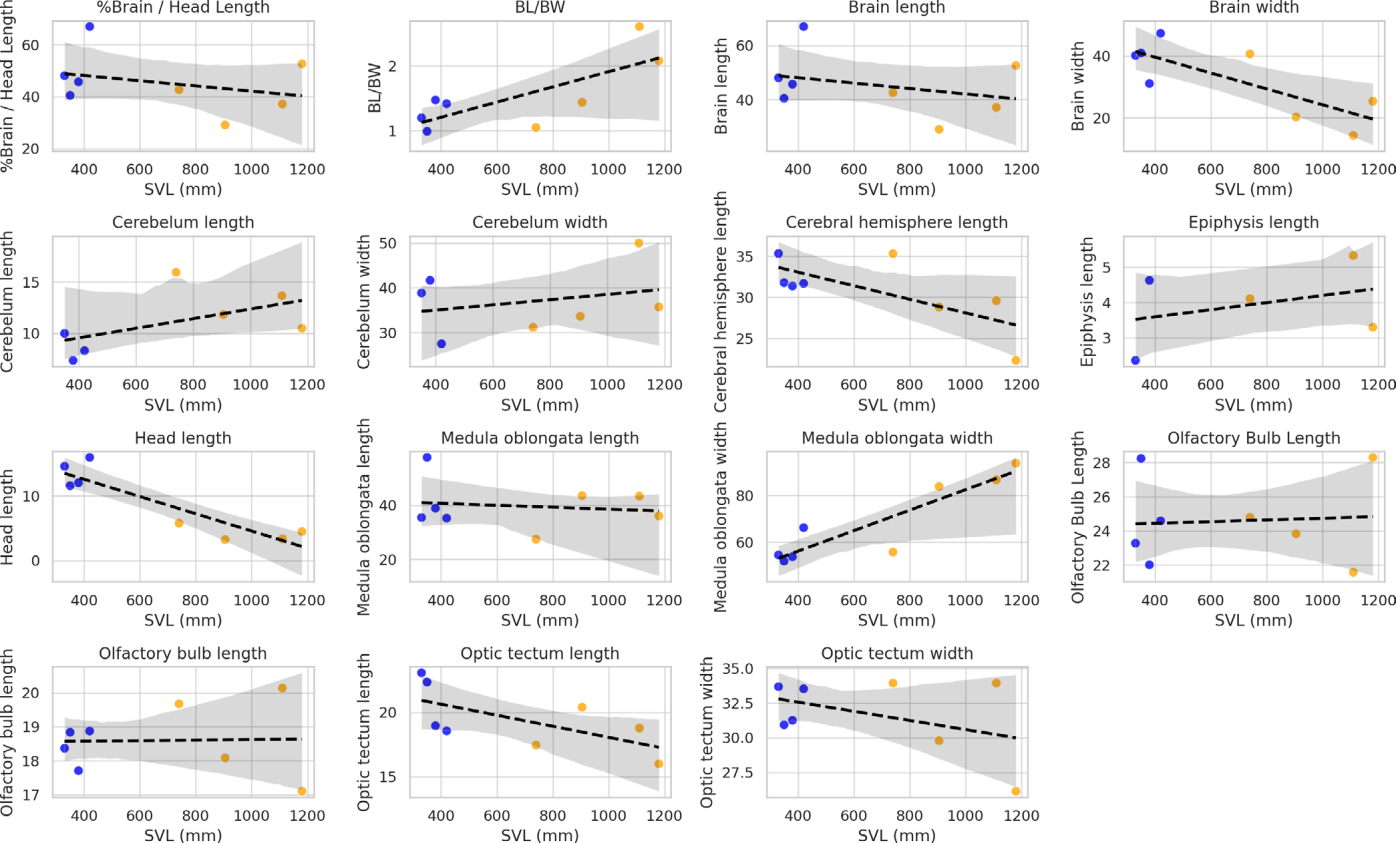
Scatter plots with fitted trend lines for brain‐related measurements as a function of snout‐vent length (SVL) for young (blue) and adult (orange) specimens of *Crotalus durissus*.

Linear regression and box plot charts indicate an evident relationship between a few brain measurements and an increase of SVL (see Figure 5), as follows. According to our results, BL in relation to HL (%Brain/HL in Figure 5), CHL, optic tectum length/width, and HL generally exhibited a negative correlation with SVL, particularly in adults, suggesting potential allometric scaling and a reduction in relative brain size with increasing body size. Conversely, the relation of BL with BW indicated that brains tend to be more elongated with an increase of SVL, with young specimens showing proportionally wider and shorter brains. The relation of CL and width, as well as medulla oblongata width, showed a positive relation with SVL increase, indicating regions that may continue growing proportionally with body size. The EL presented a minor positive trend with SVL but showed overlap between groups.

**FIGURE 6:**
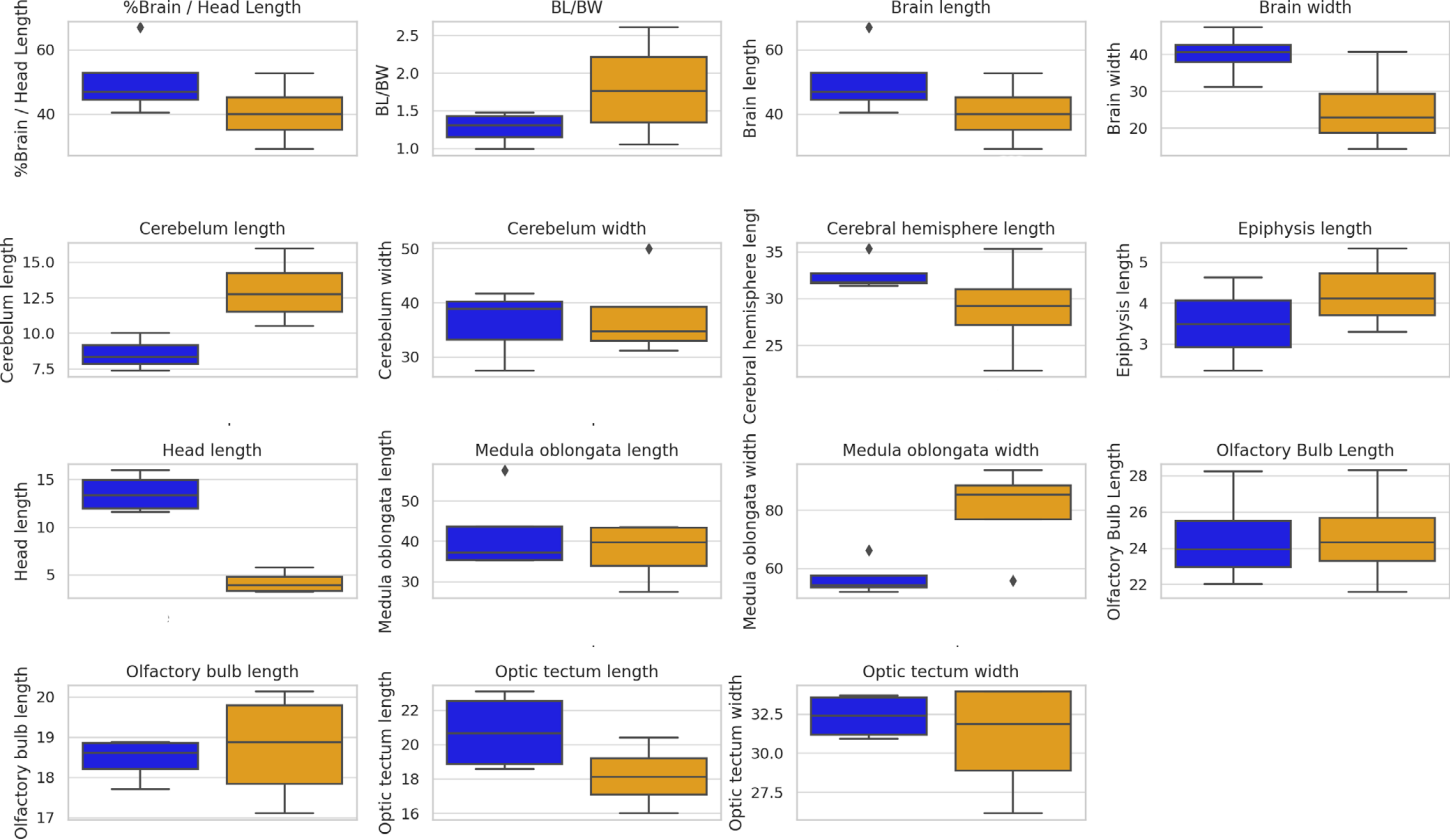
Boxplots comparing brain‐related measurements between young (blue) and adult (orange) individuals. Outliers are displayed as individual points, highlighting variability within each group.

Boxplots (Figure 6) highlighted differences between young and adults, with the latter exhibiting larger mean values for variables such as medulla oblongata width, cerebellum width, and OTW, whereas young individuals displayed narrower distributions for CHL and olfactory bulb dimensions. Notable outliers were observed in variables like medulla oblongata width, CHL, and %Brain/HL, particularly within the young group, which may reflect individual variability or methodological constraints (reduced sample).

## 4 DISCUSSION

Herein, we present the first detailed and comparative study of the macroscopic brain anatomy of *C. durissus*, providing a relevant effort on the comprehension of snake brain morphology, including data on ontogenetic variation. Even though detailed descriptive studies are lacking for several snake lineages, recent studies have provided several evolutionary insights on the variation of brain/endocranium across major snake lineages, pointing out its potential utility for systematics and evolution (e.g., Scanferla, 2022), related to locomotion, foraging habit, or activity pattern (e.g., Macri et al., 2019; Segall et al., 2021).

In terms of systematic accounts and habitat use related to brain morphology, Scanferla (2022) has provided several comparisons of “scolecophidians” (=worm snakes) and alethinophidians, including some variations of other burrowing lineages such as uropeltids. For instance, both worm snakes and uropeltids exhibit a longitudinal shortening of the brain, creating a considerable reduction of space and overlapping between brain segments, consequently affecting the different brain segments in these fossorial lineages (Scanferla, 2022; Senn, 1966). In contrast, non‐fossorial species would exhibit the opposite morphology, being linearly arranged without such an overlap, as found herein for *C. durissus*.

The elongated aspect of the brain of *C. durissus* (especially the adults) resembles those of alethinophidian species that exhibit terrestrial habits in being elongated (Scanferla, 2022; Senn, 1966). Additionally, as in other surface‐dwelling alethinophidians (as opposed to burrowing of cryptozoid specieis), the eye is well developed, generating elongated olfactory bulbs, as found for the specimens examined herein. *C. durissus* does not exhibit highly developed colliculi; consequently, it shows a more elongated optic tectum (Scanferla, 2022; present study). Since the optic tectum is associated with vision, and as the eyes tend to be large in relation to the total size of the head, the optic tectum tends to be spacious compared to the length of the brain (Scanferla, 2022). Such results are in accordance with the general habitat of the species, which is predominantly terrestrial, although the species may use elevated surfaces like rocks or low vegetation for thermoregulation or ambush hunting (Tozetti & Martins, 1993).

The morphology of the cerebral hemispheres might also vary among snake clades (Scanferla, 2022; Segall et al., 2021). In most alethinophidians these structures—as in *C. durissus* (present study)—strongly bulge laterally, contrasting with scolecophidians and fossorial/cryptozoic alethinophidians that lack such a posterior widening (Scanferla, 2022). Other similarities of the brain structures of *C. durissus* that are highly similar to surface‐dwelling species examined by Scanferla (2022) include the presence of a well‐developed and rounded pituitary gland. However, in three individuals the pituitary gland was semi‐triangular, as opposed to the pattern mentioned above. Since this represents a relatively small structure, the variation observed in shape might be related to preservation biases, most likely associated with incorrect fixation of individuals leading to shrinkage by dehydration.

The cerebellum represents one of the most important hindbrain structures present in snakes since it plays a crucial role in coordinating motor activity, including balance, posture, and locomotion; therefore, directly reflecting ecological and behavioral adaptations (Macri et al., 2019; Segall et al., 2021). This structure is fundamental in providing sensory integration and complex motor control, and previous studies have shown that the cerebellar shape is likely a more relevant feature of vertebrate brain evolution than its relative size (Macri et al., 2019; Scanferla, 2022; Segall et al., 2021). Regarding cerebellar shape, our results showed that *C. durissus* exhibited a trapezoidal cerebellum, a common pattern found in snakes in contrast to lizards (Macri et al., 2019). Macri et al. (2019) have found a direct relation with the shape and curvature of the cerebellum associated with different locomotor behaviors within snake lineages (i.e., burrowers, facultative burrowers, multi‐habitat with lateral undulation, or multi‐habit with other movements). While burrower and facultative burrower species exhibited anteriorly convex and posteriorly concave cerebella (Macri et al., 2019), the cerebellum of *C. durissus* is anteriorly convex and posteriorly concave (present study), in accordance with the general shape found for multi‐habitat species, as expected for the species. Segall et al. (2021) have also found a significant distinction in cerebellum shape across habitat use/foraging habitat. According to the author, terrestrial foragers have a laterally expanded cerebellum in its anterior part, with the posterior one being more elongated and slender. Since *C. durissus* exhibits a predominantly terrestrial lifestyle with a few records of arboreality, cerebellum shape was similar to terrestrial taxa examined by the author, suggesting that the shape of this structure might be associated with habitat use. However, general cerebellum shape was slightly variable within both juveniles and adults, and such demands additional studies since they might be a result of incorrect fixation/dehydration.

Activity period is also significantly related to brain shape—even though in smaller proportion than size—especially in the areas related to olfaction and vision (Segall et al., 2021). Thus, both olfaction and optic tracts vary when comparing diurnal, cathemeral, and nocturnal species (Segall et al., 2021). According to Segall et al. (2021), diurnal species have more elongated and bifurcated olfactory bulbs, with wide and long optic tracts. Cathemeral species exhibit the shortest bifurcation of the olfactory bulbs, while the optic tectum is of intermediate proportion compared to diurnal and nocturnal species. Nocturnal species have the shortest and bulkiest olfactory tract, with thin and slender optic tracts. Our descriptions are based on macroscopic observations, and comparisons with general endocranial shape provided by Segall et al. (2021) would be merely tentative. However, adult individuals of *C. durissus* exhibited elongated olfactory bulbs with less pronounced bifurcations and an elongated optic tectum, apparently in a similar pattern of cathemeral/nocturnal species (see Segall et al., 2021). Further detailed endocranial studies are recommended in order to confirm whether the general morphology of the species maintains a strong association to the species' habits.

No significant differences were observed in the brain or its segments between juvenile male and female individuals. However, broader studies, including adult males, are necessary to confirm whether the sexual dimorphism is absent or a limitation of the current sample. Snakes are unique among reptiles, as their braincase nearly encloses the entire brain, filling the neurocranial cavity (Scanferla, 2022). Given that several snake lineages exhibit sexual dimorphism in head and skull size and shape (e.g., Meik et al., 2012; Murta‐Fonseca et al., 2019), and considering the brain's close relationship with skull morphology, further research is needed to determine whether sexual dimorphism in brain size or shape is present. These studies could explore potential variations in brain or segment morphology that are independent of skull shape, potentially linked to ecological or behavioral differences, such as during courtship.

Previous studies (e.g., Stark & Pinchera‐donoso, 2022) have pointed out that increases in brain mass are predicted by larger body mass for both amphibians and reptiles, with body size explaining a major part of the variance in brain size in reptiles (Lara et al., 2021). Even though allometric growth seems to be relatively well conserved in snakes (Lara et al., 2021), the relative size of specimens seems to account for a huge percentage of brain shape (Segall et al., 2021). Though we did not find major differences in brain shape across different sizes, we observed scaling patterns between brain measurements and SVL, highlighting distinct ontogenetic trends across juveniles and adults. The negative correlation observed for variables such as brain length, CHL, and HL with SVL in adults suggests a potential allometric scaling, where brain size does not keep pace with increasing body size. This pattern is consistent with findings in other vertebrate groups, where relative brain size often decreases with growth due to energetic constraints or functional priorities during development (e.g., Harvey & Pagel, 1988; Heldstab et al., 2022).

On the other hand, while larger specimens exhibited smaller brains in relation to HL, they also tended to exhibit more elongated brains, while smaller individuals showed shortened and more robust brains, mainly in the olfactory bulb and cerebral hemispheres. Previous studies (Macri et al., 2019; Segall et al., 2021) have pointed out that snake species with larger SVLs would have more elongated brains, while small species would have reduced and narrow brains. Though such a variation in shape/size has previously been reported exclusively for adult individuals, we have found an elongation of the brain through ontogeny for *C. durissus*. Specific mechanisms that drive such an ontogenetic elongation are yet to be uncovered, since such a variation might be associated with a series of unknown factors yet to be investigated, such as (1) ontogenetic variation (=elongation) possibly being directly related to skull ontogenetic allometry, as observed in other viperid and alethinophidian species (e.g., Carrasco et al., 2023; Murta‐Fonseca et al., 2019); (2) brain variation being associated with minor changes in locomotion and diet habits throughout ontogeny, directly affecting sensorial brain segments (Segall et al., 2021); or other factors yet to be tested.

Other brain elements showed an indication of positive or negative association with SVL length, and also need further examinations, as for CL/width, CHL, medulla oblongata width, and OTW. These positive trends suggest that specific brain regions may continue growing proportionally with body size (Barton & Harvey, 2000), and these findings might be explained by the fact that sensory and motor‐related brain regions might adapt to accommodate increased ecological demands or behaviors associated with larger body sizes (Striedter, 2005; Hoops et al. 2017). Additionally, relatively stable patterns observed in variables such as OTL and width, with minor variability among adults, may reflect the relative independence of these regions from allometric influences or their functional stabilization during development (Zimmermann et al. 2011). Additionally, adults exhibited larger mean values for medulla oblongata width, cerebellum width, and OTW, likely reflecting the functional demands of maturity, such as enhanced motor coordination and sensory integration (Yopak et al., 2010). In contrast, juveniles displayed narrower distributions in CHL and olfactory bulb dimensions, potentially reflecting a developmental stage of rapid growth or variability in individual ontogenetic trajectories.

Since different regions of the brain showed different patterns of allometric growth, these patterns resonate with possible modular growth, since brain modularity may provide a basis for adaptability in the evolution of vertebrates (Redies & Puelles, 2001; Tsuboi et al., 2018; Sansalone et al., 2020). However, a key limitation of this study lies in the small sample size, which restricts the generalizability of these findings and reduces the statistical power to detect subtle patterns. Future studies with a larger and more diverse sample could allow for the inclusion of robust statistical tests, such as multivariate regression models, to better quantify the relative influence of SVL and group on brain morphology. Additionally, incorporating 3D morphometric techniques or imaging‐based volumetric analyses could provide a more comprehensive understanding of developmental changes and inter‐individual variability. In such a sense, it is crucial to consider the significant variation in proportional brain volume within the endocranium across different developmental stages (ontogeny). This aspect should be controlled for interspecific studies (Watanabe et al., 2019) since most of the studies of brain variation and evolution have been based on endocranium endocasts. Surprisingly, this particular aspect has not received much attention in previous neuroanatomical studies of snakes.

## 5 CONCLUSIONS

In this study, we provide the first detailed description of *C. durissus* brain, providing valuable insights into ontogenetic brain variation in snakes. The macroscopic features identified in this species suggest a brain structure typical of a multi‐habitat and terrestrial alethinophidian species, contributing to the understanding of brain morphological evolution in snakes. Our findings revealed distinct scaling patterns in brain measurements with SVL, with variables such as BL and CHL decreasing with SVL, while others like OBL and medulla oblongata width increased. These patterns highlight the complex interplay between brain growth, body size, and functional specialization.

Significant differences were also observed between young and adults, with adults exhibiting larger mean values in cerebellum and medulla oblongata measurements. These differences emphasize the importance of considering developmental stages when studying brain morphology. However, the small sample size limits the generalizability of these findings and underscores the need for future studies with larger and more diverse datasets. Expanding this research to include a broader range of species and utilizing advanced methodologies, such as 3D morphometric analyses and volumetric imaging, is critical for validating these patterns and exploring their taxonomic and systematic implications. Continued investigation in this area will enhance our understanding of brain allometry and its evolutionary significance within snakes.

## AUTHOR CONTRIBUTIONS

**Giordanna Issa Lucas:** Conceptualization; investigation; writing – original draft; methodology; validation; visualization; writing – review and editing. **Angele Martins:** Writing – original draft; writing – review and editing; funding acquisition; project administration; supervision; resources; data curation.
